# Protecting Police Officers Against Burnout: Overcoming a Fragmented Research Field

**DOI:** 10.1007/s11896-023-09584-4

**Published:** 2023-04-06

**Authors:** Isabel Correia, Ângela Romão, Andreia E. Almeida, Sara Ramos

**Affiliations:** 1 Iscte-Instituto Universitário de Lisboa, CIS-Iscte, Lisboa, Portugal; 2 Iscte -Instituto Universitário de Lisboa, DINÂMIA’CET-Iscte, Lisboa, Portugal

**Keywords:** Burnout, Organizational justice, Organizational identification, Psychosocial risk factors, Police officers, Guarda Nacional Republicana (GNR)

## Abstract

This study aims to identify the determinants of burnout in police officers. We considered a wide range of psychosocial risk factors, individual variables that have been previously found to be associated with burnout in police officers (affective and cognitive empathy, self-care), and variables whose unique impact on burnout of police officers needs further clarification (organizational justice and organizational identification). The study was conducted in Portugal, and the sample was constituted by 573 members of the National Republican Guard (GNR—Guarda Nacional Republicana). The participants were invited to answer an online anonymous survey, which included previously validated measures of the following variables: burnout (exhaustion and disengagement), psychosocial risk factors, self-care, empathy (cognitive and affective), organizational justice, and organizational identification. Furthermore, we controlled for the potential impact of demographic variables (age, gender, years of professional experience, religiosity, political orientation, and income). Multiple regression analysis showed that when taken together, only a few of the variables associated with burnout had a unique impact on both exhaustion and disengagement: quantitative demands and affective empathy were burnout risk factors; meaningful work, organizational justice (distributive justice, procedural justice, and interactional justice), and organizational identification were burnout protective factors. Our results highlight the importance of developing theoretical models and planning interventions to prevent burnout in police officers, focusing mainly on the above-mentioned variables.

## Introduction

Police officers’ work is characterized by high demands and potentially chronic stressors. In their everyday activity, police officers face criminals and violence and victims in great suffering, and they may even put their lives at risk to protect others (e.g. Gomes and Afonso 2016). Moreover, this is very often done with low human and material resources, heavy bureaucratic duties, low legitimacy from the population, and lack of support from family and friends (see Burke and Mikkelsen 2006). This exposure to ongoing occupational stressors, such as the preceding, leads to high levels of burnout and mental health problems in police officers of many countries (Purba and Demou 2019; Queirós et al. 2020, for reviews), including Portugal in which the present study has been conducted (Afonso and Gomes 2009; Rosa et al. 2015).

Burnout is a psychological syndrome that may seriously affect employees, their employers, and clients (Freudenberger 1974; Leiter and Maslach 2003; Maslach 1976). One possible way of reducing burnout is to reduce risk factors and increase protective factors (e.g. Xanthopoulou et al. 2007). It is therefore important that research explores the risk and protective factors that may defend against burnout in police officers. However, so far, most studies (e.g. Santa Maria et al. 2021) only focused on a small number of potential predictors of burnout. In our opinion, this might not be the best way to advance research because the identification of the strongest determinants of burnout on which causal models with a high explanatory potential might be built is still lacking. As a matter of fact, although many variables may indeed be associated with burnout, it is possible that we find that they lose their impact when other potential predictors are taken into account.

In this research, we intend to fill this gap and conduct a thorough investigation of risk and protective factors for burnout in a single study aiming to identify the most important determinants for burnout in police officers. We included a wide range of psychosocial risk factors, some individual variables that have been previously found to be associated with burnout (self-care and cognitive and affective empathy), and two potentially important burnout protective factors for police officers, whose unique impact on burnout has not yet been sufficiently addressed (organizational justice and organizational identification).

### Burnout

Burnout was originally characterized by 3 dimensions: exhaustion, cynicism, and inefficacy (Leiter and Maslach 2003; Maslach 1976). Subsequently, professional inefficacy was found to have little connection with the two other burnout components or with antecedents and outcomes (Lee and Ashforth 1996). In line with this finding, the present study used a two-dimensional measure of burnout: the Oldenburg Burnout Inventory (OLBI; Demerouti et al. 2001) that measures exhaustion and disengagement from work. According to Demerouti et al. (2003), exhaustion is an extreme form of fatigue resulting from a prolonged and intense physical, affective, and cognitive strain caused by chronic exposure to adverse working conditions. Disengagement is distancing oneself from one’s work and represents an extensive and intensive reaction in terms of an emotional, cognitive, and behavioural work rejection. The OLBI’s concept of disengagement is broader than that of depersonalization on MBI’s, which is more focused on cynical or excessively detached responses to others in the work context.

The Maslach Burnout Inventory (MBI; Maslach et al. 2016) is commercially available, and it is the most used measure of burnout (Sinval et al. 2019). However, OLBI has been considered a prominent alternative to MBI (Sinval et al. 2019), and its use is free. For this reason, we decided to use it in this study to measure burnout.

### Correlates of Burnout in Police Officers

In police officers, burnout has been shown to negatively affect physical health (e.g. metabolic syndromes, Hartley et al. 2012) and mental health (e.g. depression, Hakanen et al. 2008; anxiety, Santa Maria et al. 2018; and a high-risk of suicide, Costa et al. 2019; Violanti et al. 2019). Burnout also negatively impacts on the quality of the services police officers provide (e.g. performance deficits, Lerner and Henke 2008) and on the police organization for which they work (e.g. counterproductive work behaviour, Smoktunowicz et al. 2015; or sickness absence, Magnavita and Garbarino 2013; and increased turnover intent, Yun et al. 2015).

Moreover, burnout may also negatively affect the legitimacy of the social system itself, because police officers are representatives of the social system (Tyler et al. 2015) and they are armed (Tyler et al. 2007). It has been shown that burnout in police officers is correlated with more positive attitudes towards the use of force and frequent use of violence during officers’ duty (Kop and Euwema 2001) and aggressivity (Queirós et al. 2013).

### Psychosocial Risk Factors and Burnout

Psychosocial risk factors are the organizational and psychosocial factors of organizations that can lead to maladaptation, tension, or psychophysiological stress responses and are likely to negatively affect health (e.g. Talavera-Velasco et al. 2018). Because of their centrality for work environment issues, psychosocial risk factors have been the object of much research, some of which has also been conducted to find out the impact of psychosocial risk factors on burnout in police officers.

Some of these factors have been found to have a significant impact on police officer burnout, namely high quantitative demands, high emotional demands, and high work family conflict (Burke 1988; Burke and Mikkelsen 2006; Hall et al. 2010; Martinussen et al. 2007), low social support and low sense of community (Burke and Mikkelsen 2006; Smoktunowicz et al. 2015), lack of job control (Bhowmick and Mulla 2021), low job rewards (Santa Maria et al. 2021), low work importance and significance (Pines and Keinan 2005), and shift work (Cheng and Cheng 2016; Wisetborisut et al. 2011). The present study intends to contribute to the better understanding of the relation between psychosocial risk factors and burnout in police officers.

### Self-care and Burnout

According to the World Health Organization (1983), “self-care in health refers to the activities individuals, families and communities undertake with the intention of enhancing health, preventing disease, limiting illness, and restoring health” (p. 181). Three dimensions of mental health have been distinguished: physical, inner, and social self-care (e.g. Galiana et al. 2015): physical care refers to the incorporation of physical activities in daily life; inner care is related with caring for the self’s inner world; and social care comes both from personal (family and friends) and professional systems. These three dimensions of self-care have already been shown to protect against stress in several occupations (Burnett et al. 2019; Myers et al. 2012). However, little research has investigated the impact of self-care practices on police employees: physical exercise has been linked to improved psychological health within a policing context (Gerber et al. 2010), and mental self-care has significantly predicted burnout in police officers (Burnett et al. 2019). These results suggest that self-care may be an important protector of well-being in police officers.

### Empathy and Burnout

Empathy is generally considered to be a two-dimensional construct with both an affective and cognitive dimension (Mehrabian 1997). The affective dimension refers to the capability to share another person’s emotional state (Eisenberg and Strayer 1987), while the cognitive dimension refers to the ability to understand (but not necessarily share) another’s emotional state (Davis 1994).

There has been some controversy about the protective or risk role of empathy for burnout of professionals whose work involves contact with people. Although empathy is an important component in providing effective care and therefore may protect against burnout, it can also create vulnerability for stress-related conditions (Zenasni et al. 2012). For example, in health care workers, the results are not conclusive. Some studies found empathy to be protective against burnout (see Wilkinson et al. 2017, for a review), while others found it—namely affective empathy—a risk factor (Correia and Almeida 2020; Varela and Correia 2023).

With police officers, the evidence is also not entirely clear: empathy has been found to be a risk factor for mental health (Beagley et al. 2018), but Losung et al. (2021) found it protective against burnout. Our study aims to contribute to the understanding of affective and cognitive empathy’s role in this field.

### Organizational Justice and Burnout

The perception of justice has been acknowledged as a core human need that when unmet produces a decrease in well-being (Lerner 1980). Police officer perceptions of justice, namely organizational justice, have been shown to be an important predictor of police officers’ attitudes towards the public and community (e.g. Bradford et al. 2013; Carr and Maxwell 2018; Trinkner and Tyler 2016), as well as rule adherence and compliance with supervisor directives (Haas et al. 2015; Tyler et al. 2007).

Organizational justice (distributive, procedural, and interactional) has also been identified as an important predictor of burnout (Maslach et al. 2001). Distributive justice refers to the perception as to whether the resources allocated to people are “deserved” or not, according to their contributions (Adams 1965); procedural justice refers to the fairness of the means by which distributions, or decisions about them, are made (Leventhal 1980; Thibaut and Walker 1975); and interactional justice refers to the respectful and proper manner by which authorities communicate procedural details and justify their decisions using honest and truthful information (Bies and Moag 1986). For interactional justice, two components have been further distinguished (Greenberg 1993): interpersonal justice (being treated with politeness, dignity, and respect) and informational justice (being adequately informed about the reasons behind decisions, expectations, and processes). As there is still some conceptual disagreement about the distinctiveness of these constructs—particularly procedural, interpersonal, and informational justice (e.g. Carr and Maxwell 2018)—and the scale of organizational justice that we used (Rego 2000) does not distinguish between interpersonal and informational justice, the present study considers a three-dimensional model of organizational justice.

A significant correlation between an overall measure of organizational justice and an overall measure of burnout has been found in police officers (Kaygusuz and Beduk 2015). In other occupations, some studies have assessed and compared the unique impact of these three dimensions of organizational justice in burnout. For example, in a study with hotel employees that had regular contact with customers as part of their work, Moliner et al. (2005) found that distributive, procedural, and interactional justice were all negatively associated with both cynicism and exhaustion; but when considered together, only procedural justice was a significant predictor of both dimensions of burnout. Another study with physicians and nurses (Correia and Almeida 2020) found that procedural justice, distributive justice, and justice of colleagues were negatively associated with both dimensions of burnout. However, in that same study, only procedural justice combined with interactional justice had a unique impact on the two dimensions of burnout in physicians and nurses.

Our study includes other dimensions of justice that we think are particularly relevant for police officers, such as justice of colleagues and justice of citizens. Previous studies on physicians (Smets et al. 2004) showed that perceived injustice from colleagues was associated with exhaustion, and perceived injustice from patients was associated with both exhaustion and depersonalization.

### Organizational Identification and Burnout

According to Social Identity Theory (Tajfel 1978), individuals belong to various groups, and these groups define different social identities. Social identification is the strength of the sense of membership within a social group (Turner et al. 1987). Although the relation between group identification and well-being seems to be bidirectional (Delvaux et al. 2015), either mutually reinforcing or mutually dampening each other over time, the majority of research has focused on the impact of group identification on well-being (e.g. Jetten et al. 2012).

Given that it fills a considerable amount of most people’s time, work has substantial implications for identity, either as organizational identification (Mael and Ashforth 1992) or professional identification (Lammers et al. 2013). This implies that individuals define themselves, respectively, in terms of the organization in which they are a member or the professional activity they perform.

Identification as a police officer is associated with better performance (DeCarufel and Schaan 1990; Diefendorff et al. 2002; Lambert et al. 2019), more supportive attitudes towards progressive policing, values, greater likelihood of whistle-blowing behaviour, and lower corruption (Haarr 1997; Lambert et al. 2019). There is less evidence about the association of professional identification and burnout. In other professional groups, a strong professional identification has been shown to be an important burnout protector (Avanzi et al. 2018; Correia and Almeida 2020; Varela and Correia 2023), but for police officers, the evidence is less clear. Schaible (2018) showed that officer identification has benefits early in the career, especially when supported by congruent values, but not in later stages.

As in Portugal police officers usually spend their entire career in only one police force where they can perform different functions throughout their working life, we have considered a measure of identification with the organization instead of a measure of identification with the profession.

### Demographic Variables

We controlled for several variables that may affect the proposed relationships but that were not of direct theoretical interest. As far as gender is concerned, Schaufeli and Enzmann (1998) found that female officers had significantly elevated levels of burnout, but McCarty et al. (2007) and Tsai et al. (2018) found no significant differences in measures of burnout across genders.

There are equally conflicting results with regard to years of professional experience. Burke (1989) found that police officers with more years of professional experience had reported greater stress, burnout, and more sick days over the previous 6 months than those who had fewer years of experience. This may be because the negative effects of service had accumulated over an extended period of time.

Income was also included because it is an important predictor of well-being (Lucas and Schimmack 2009). We also controlled for religion and political orientation of the participants, because religion is another important predictor of well-being (Koenig 2012) and is usually associated with a more right-wing political orientation (Correia et al. 2018). Finally, questions related with the change of functions because of the COVID-19 pandemic, and that might have been additional stressors for police officers, were also included as control variables.

## Overview and Hypothesis

Our research explores the unique impact of a wide range of psychosocial risk factors and individual variables on police officer burnout. Generally, for each factor taken in isolation, we hope to replicate the same pattern of findings of previous research (already presented in the revision of literature section of this manuscript), but when all the factors are considered together, it will be possible to find which of them are the most impactful. Indeed, considering all these factors simultaneously is fundamental for further interventions aiming to prevent burnout in the police officer sector.

The present study was conducted with police officers belonging to the Portuguese Republican National Guard (Guarda Nacional Republicana). The police officers of this security force have military and police training and are organized in a special core of troops with the mission of maintaining public order and the security of people and goods (https://www.gnr.pt/missao.aspx). Previous studies to measure burnout or occupational stress among Portuguese police officers (from the Portuguese Republican National Guard, GNR, Afonso and Gomes 2009; and also from the Portuguese National Police, PSP, Queirós et al. 2020) showed moderate to critical values of burnout and stress.

## Method

### Procedure

This study was carried out in accordance with the recommendations of the Ethics Guidelines of the Scientific Commission of the Research Centre in which the study was conducted and with the Declaration of Helsinki.

According to the Ethics Guidelines of the Scientific Commission of the Research Centre in which the study was conducted, the study is exempt from formal ethical approval because the study includes only surveys collected anonymously and without obligation to complete, it does not involve participants from a population of concern, it does not involve questions about undesirable personal characteristics nor potentially endangering information, it does not involve deception, it does not require participants to ingest any substance, and it does not involve any invasive measure.

An online survey was created using Qualtrics® (Qualtrics, Inc.; Provo, UT, USA), and the weblink was disseminated by one of the Trade Unions of the National Republican Guard in Portugal (APG/GNR—Associação dos Profissionais da Guarda) through their official webpages and social networks and also to GNR professionals that are not members of that union.

Participants were informed that participation was voluntary and anonymous. They were also provided with information about the general purpose of the study. They were told that the study was non-invasive and that there were no physical, financial, social, legal, or other risks connected with it. It was also explained that they could withdraw from the study by closing the web browser. Contact information for the research team was provided to all participants in case they wished to obtain additional information or had any questions about the study. Informed consent was obtained from all individual participants included in the study. After informed consent and agreement to participate were obtained, participants were then presented with the survey. At the end, the participants were thanked for their participation and debriefed, and the research team contact information was again provided.

Data collection took place between March and April 2021 during the COVID-19 pandemic.

### Participants

The sample of the present study was composed of 573 members of the Guarda Nacional Republicana, aged between 20 and 65 years old (*M* = 40.03, *SD* = 7.95), and 90.2% were male. The average number of years that participants had exercised their profession was around 17 (SD = 8.69). Most of them (75.6%) had 10 to 12 years of education, were married, or living in a consensual union (72.6%) and had children (75.4%). The number of hours worked per week were about 42 (SD = 9.96). The majority reported that they did shift work (83.8%), their professional category was Guard (86.4%), and they had operational functions (74.9%). As far as income was concerned, 42.2% of the participants considered their present income as being enough to live, and 38.2% stated that it was difficult to live with their present income. The participants were from all regions of Portugal but mostly from the Centre (31.9%) and North (26.7%), and most of them (82.9%) were not displaced from their family. From the whole sample, 65.8% of participants reported that the COVID-19 pandemic had not changed the functions they were due to perform. Table 1 presents the descriptive statistics of socio-demographic variables.

**Table 1:** Number of items, Cronbach’s alphas, and descriptive statistics of variables

	**Items**	**α**	**Mean**	**SD**
**Demographic and control variables**				
Work hours per week	1	---	42.35	9.96
Years of professional experience	1	---	17.32	8.69
Sex	1			
Male (%)		---	90.2	
Female (%)			8.1	
Not referred (%)			1.7	
Age (M/SD)	1	---	40.03	7.95
Area of residence	1			
North (%)			26.7	
Centre (%)			31.9	
South (%)			16.2	
Metropolitan area Lisbon (%)			18.7	
Metropolitan area Oporto (%)			3.0	
Azores/Madeira Island (%)			3.5	
Other countries (%)			-	
Income	1	---	2.32	0.78
Religiosity	1	---	2.54	1.06
Political orientation	1	---	2.48	1.32
Displaced from the family	1	---		
Yes (%)			17.1	
No (%)			82.9	
Change of functions due to COVID-19	1	---		
Yes (%)			34.2	
No (%)			65.8	
Shift work	1	---		
Yes (%)			83.8	
No (%)			16.2	
**Demands at work (COPSOQ - II)**				
Quantitative demands	3	.76	2.99	0.76
Work pace	1	---	3.18	0.88
Emotional and cognitive demands	4	.65	3.76	0.64
**Work organization and job content (COPSOQ - II)**				
Influence at work	4	.72	2.65	0.79
Possibilities for development	3	.69	3.69	0.70
Meaning of work	3	.79	3.58	0.77
Commitment to work	2	.51	2.82	0.86
**Social relationships and leadership (COPSOQ - II)**				
Predictability	2	.74	2.64	0.80
Rewards	3	.86	3.33	0.92
Role-clarity	3	.68	3.78	0.68
Quality of leadership	4	.91	2.80	0.90
Social support from supervisor	3	.86	2.70	0.90
Social support from colleagues	3	.77	3.36	0.71
**Values at the workplace (COPSOQ - II)**				
Trust vertical	3	.81	3.43	0.82
Trust horizontal	3	.76	2.69	0.75
Justice and respect	3	.81	3.04	0.82
Social community at work	3	.88	3.79	0.79
**Other variables**				
Exhaustion	8	.90	3.25	0.80
Disengagement	8	.89	3.33	0.86
Self-care	9	.83	3.19	0.73
Affective empathy	3	.85	2.64	0.82
Cognitive empathy	4	.78	3.79	0.48
Distributive justice	5	.87	1.81	0.76
Procedural justice	4	.79	2.04	0.76
Interactional justice	5	.95	3.06	1.05
Justice from colleagues	2	.91	3.59	0.75
Justice from citizens	2	.94	3.41	0.98
Organizational identification	1	---	3.43	1.07

For all variables with two items, the measure of internal consistency is Spearman-Brown, instead of Cronbach Alpha

### Measures

#### Burnout

We used the Portuguese adaption (e.g. Sinval et al. 2019) of the Oldenburg Burnout Inventory (OLBI, Bakker et al. 2004). The OLBI consists of two dimensions with eight items each: exhaustion (e.g. “There are days when I feel tired before I arrive at work”, *α* = 0.90 and disengagement (e.g. “It happens more and more often that I talk about my work in a negative way”, *α* = 0.89). Responses were given on a 5-point Likert scale, 1 = “totally disagree” to 5 = “totally agree”.

#### Psychosocial Risk Factors

We measured work psychosocial risk factors, with the Portuguese version of the Copenhagen Psychosocial Questionnaire (COPSOQ-II; Silva et al. 2012). COPSOQ is well-established (Kristensen et al. 2005) and has already been translated into more than 20 languages. The Portuguese version of the COPSOQ-II evaluates different psychosocial factors at work, health, and work-related outcomes and consists of 29 scales classified in eight thematic domains: demands at work; work organization and job content; social relationships and leadership; work-individual interface; values at the workplace, personality, health, and well-being; and offensive behaviours. For this study, we considered the items of four thematic domains: demands at work, work organization and job content, social relationships and leadership, and values at the workplace.^1^ The number of items per scale is presented in Table 1. All items were scored with a 5-point Likert scale ranging from 1 (“never”) to 5 (“always”), except for the work-individual interface items where the categories ranged from 1 (“nothing”) to 5 (“always”). The total score on a scale was the mean of the scores of the individual items.

Additionally, we asked participants if they did or did not do shift work, in a 1 (“yes”) or 2 (“no”) response format.

#### Self-care

We used the Professional Self-Care Scale (Galiana et al. 2015), which has nine items and was answered on a 5-point Likert scale ranging from 1 (“totally disagree”) to 5 (“totally agree”) and assesses professionals’ self-care in three areas: physical self-care, inner self-care, and social self-care that can be combined to create an overall factor of self-care. Examples of these questions are “I do exercise regularly” (physical self-care dimension); “My self-care includes getting involved in spiritual practice via meditation, prayer, other mindful practice” (inner self-care); or “I believe that my family relationships are satisfactory” (social self-care).

However, in our sample, a principal component analysis (either with varimax or oblimin rotation) did not replicate the three-factor structure, and therefore we aggregated the items in one single factor of self-care that has a very good internal consistency, *α* = 0.83.

#### Empathy

Empathy was measured using the Portuguese Adaptation of the Basic Empathy Scale short version (BES-A) (Pechorro et al. 2018). This version is a translation and validation of a shorter version (Salas-Wright et al. 2012) of the Basic Empathy Scale (BES) (Jolliffe and Farrington 2006). This BES-A version has seven items, with three items for the affective dimension (e.g. “After being with a friend who is sad about something, I usually feel sad”, *α* = 0.85) and four items for the cognitive dimension (e.g. “I can often understand how people are feeling even before they tell me”, *α* = 0.78). The items were answered on a 5-point Likert response scale ranging from 1 (“totally disagree”) to 5 (“totally agree”).

#### Organizational Justice Perceptions

Distributive, procedural, and interactional justice were measured using items taken from the Organizational Justice Scale (Rego 2000). Distributive justice was assessed with five items (e.g. “In general, the rewards I receive are fair”, *α* = 0.87); procedural justice was assessed with four items (e.g. “My institution has a mechanism that allows employees to appeal decisions”, *α* = 0.79); and interactional justice was assessed with five items (e.g. “My Commander^2^ treats me with respect and consideration”, *α* = 0.95).

Justice from colleagues was measured using two items (e.g. “My colleagues appreciate my work”, *S*_B_ = 0.91). Justice from citizens was measured using two items (“The citizens recognize my work”, *S*_B_ = 0.94). The items were answered in a 5-point Likert scale ranging from 1 (“totally disagree”) to 5 (“totally agree”). The measures of justice from colleagues and justice from citizens were taken from Correia and Almeida (2020).

#### Organizational Identification

This construct was measured with a previously validated one-item measure (“I identify with the organization I work for”; Postmes et al. 2012), and the responses were given on a 5-point Likert scale ranging from 1 (“totally disagree”) to 5 (“totally agree”). The use of single-item validated measures of organizational constructs has been increasingly accepted (Matthews et al. 2022).

#### Demographic Variables

Income was measured with an item adapted from the European Social Survey (2018): “Which of the following descriptions is closest to your current income?”, with a 4 statement response scale: 1 = “It is very difficult to live with my current income”; 2 = “It is difficult to live on my current income”; 3 = “My current income is enough to live”; 4 = “My current income allows me to live comfortably”.

Religiosity was measured with an item adapted from the European Social Survey (2018): “Regardless of whether you belong to a particular religion, how religious would you say you are”? with a 5-point Likert scale ranging from 1 (“not religious at all”) to 5 (“very religious”).

Political orientation was measured with an item adapted from the European Social Survey (2018): “In politics, people sometimes talk of “left” and “right”. Where would you place yourself on this scale, where 1 means the left and 5 means the right”?

One item used specific factors related to the COVID-19 pandemic: “Has the COVID-19 pandemic changed your functions?” This item had a Yes/No answer.

### Analytical Strategy

Data was analysed using SPSS (IBM SPSS; version 25). Firstly, a descriptive analysis was undertaken to determine characteristics of the study population and of the variables measured (Table 1). After that, Pearson’s correlation was calculated between all the measures (Tables 2 and 3). Afterwards, multiple regression analyses were carried out. Each of the burnout dimensions (exhaustion and disengagement) was treated as a dependent variable and demographic, individual, and psychosocial factors at work were used as the independent variables. The variables were only entered in the regression if they significantly correlated with one of the burnout dimensions. An alpha level of *p* < 0.05 was used for tests of statistical significance.

**Table 2:** Correlations between psychosocial risk factors, justice perceptions, and organizational identification (*N* = 573)

	1	2	3	4	5	6	7	8	9	10	11	12	13	14	15	16	17	18	19	20	21	22
1. Exhaustion	-																					
2. Disengagement	.67***	-																				
3. Quantitative demands	.48***	.31***	-																			
4. Emotional and cognitive demands	.21***	.07	.37***	-																		
5. Influence at work	− .18***	−.20***	.04	.31***	-																	
6. Possibilities for development	− .19***	−.34***	−.01	.44***	.40***	-																
7. Predictability	− 39***	−.43***	−.32***	−.10*	.27***	.26***	-															
8. Role-clarity	− .30***	−.32***	−.15**	.16***	.25***	.41***	.47***	-														
9. Meaning of work	− .42***	−.60***	−.13**	.17***	.23***	.42***	.26***	.34***	-													
10. Work pace	.37***	.24***	.64***	.41***	.08*	.07	−.23***	−.10*	−.09*	-												
11. Rewards	− .49***	−.50***	−.34***	−.04	.25***	.33***	.52***	.41***	.37***	−.22***	-											
12. Social support from supervisor	− .27***	−.41***	−.11*	.08*	.28***	.26***	.46***	.34***	.24***	−.07	.65***	-										
13. Justice and respect	− .44***	−.48***	−.38***	−.09*	.20***	.26***	.55***	.42***	.36***	−.27***	.76***	.58***	-									
14. Quality of leadership	− .38***	−.49***	−.31***	−.06	.18***	.25***	.55***	.38***	.33***	−.20***	.75***	.71***	.80***	-								
15. Trust regarding management	−.40***	−.48***	−.29***	−.01	.17***	.32***	.53***	.44***	.41***	−.21***	.72***	.54***	.76***	.73***	-							
16. Social support from colleagues	−.20***	−.19***	−.09*	.19***	.26***	.25***	.19***	.23***	.22***	−.04	.29***	.29***	.27***	.22***	.28***	-						
17. Social community at work	−.27***	−.30***	−.16***	.08	.20***	.30***	.24***	.31***	.34***	−.10*	.39***	.25***	.42***	.33***	.43***	.59***	-					
18. Distributive justice	−.38***	−.40***	−.28***	−.22***	.16***	.14**	.32***	.17***	.19***	−.28***	.33***	.25***	.35***	.33***	.26***	.07	.10*	-				
19. Procedural justice	−.40***	−.45***	−.26***	−.17***	.10*	.15**	.43***	.21***	.26***	−.23***	.33***	.27***	.40***	.36***	.31***	.07	.15***	.55***	-			
20. Interactional justice	−.40***	−.46***	−.24***	−.04	.12**	.23***	.43***	.31***	.31***	−.15***	.75***	.63***	.70***	.78***	.69***	.16***	.29***	.26***	.30***	-		
21. Justice of colleagues	−.19***	−.24***	−.06	.17***	.19***	.26***	.11**	.22***	.27***	−.02	.21***	.11**	.16***	.13**	.22***	.49***	.49***	.01	.04	.16***	-	
22. Justice of citizens	−.19***	−.18***	−.06	.05	.16***	.12**	−.19***	.11**	.20***	−.09*	.11**	.13**	.11**	.13**	.09*	.13**	.13**	.16***	.14**	.08	.24***	-
23. Organizational identification	−.41***	−.53***	−.14**	.01	.19***	.26***	.29***	.27***	.49***	−.12**	.37***	.23***	.37***	.33***	.39***	.15***	.26***	.30***	.37***	.31***	.15***	.16***

^*^p < .05; **p < .01; ***p < .001

**Table 3:** Correlations between variables (*N* = 573)

	1	2	3	4	5	6	7	8	9	10	11	12	13	14	15	16	17	18	19	20	21	22	23	24	25	26	27
1. Exhaustion	-																										
2. Disengagement	.67***	-																									
3. Age	.08	.04	-																								
4. Work hours per week	.01	−.01	−.07	-																							
5. Years of professional experience	.08*	.01	.88***	−.08*	-																						
6. Sex	.02	−.03	−.08*	−.08	−.04	-																					
7. Displaced from the family	−.03	.03	.22***	−.08	.19***	.07	-																				
8. Income	−.26***	−.22***	.01	−.11**	.03	.08	.12**	-																			
9. Shift work	−.12**	−.15***	.16***	−.03	.16***	.11*	.06	.09*	-																		
10. Quantitative demands	.48***	.31***	−.02	.16***	−.03	−.05	−.05	−.24***	−.09*	-																	
11. Emotional and cognitive demands	.21***	.07	−.10*	.10*	−.08*	−.03	−.09*	−.22***	−.10*	.37***	-																
12. Influence at work	−.18***	−.20***	.04	.11*	.06	−.07	−.00	−.02	.09*	.04	.31***	-															
13. Possibilities for development	−.19***	−.34***	.02	.05	.04	−.05	−.01	.04	−.01	−.01	.44***	.40***	-														
14. Predictability	−.39***	−.43***	.09*	−.00	.11**	−.01	.07	.15**	.13**	−.32***	−.10*	.27***	.26***	-													
15. Role-clarity	−.30***	−.33***	.12**	.02	.11**	−.03	.12**	.06	.09*	−.15***	.16***	.25***	.41***	.47***	-												
16. Meaning of work	−.42***	−.60***	−.07	.04	−.04	.06	.04	.09*	.11**	−.12**	.17***	.23***	.42***	.26***	.34***	-											
17. Work pace	.37***	.24***	−.04	.19***	−.08	−.03	−.07	−.24***	−.05	.64***	.41***	.08*	.07	−.23***	−.10*	−.09*	-										
18. Affective empathy	.26***	.15***	.19***	−.01	.19***	.01	.05	−.02	.05	.13**	−.05	−.05	−.10*	−.05	−.10*	−.10*	.07	-									
19. Cognitive empathy	.02	.06	−.04	.06	−.04	.09*	.05	−.06	−.01	.07	.25***	.14**	.20***	−.00	.11*	.13**	.16***	.01	-								
20. Self-care	−.43***	−.32***	−.03	−.12**	−.03	.14**	.04	.19***	.06	−.24***	−.08*	.08*	.12**	.24***	.22***	.30***	−.22***	−.08	.14**	-							
21. Religiosity	.01	−.10*	.16***	−.04	.13**	.05	−.01	−.03	.02	−.03	.00	.02	.04	.04	.08*	.09*	−.06	.13**	.03	.18***	-						
22. Political orientation	.11**	.02	.01	.03	.01	.03	−.06	−.14**	−.10*	.04	.07	−.03	−.01	−.03	.06	−.07	−.01	.02	−.04	−.10*	.00	-					
23. Distributive justice	−.38***	−.40***	.17***	−.07	.20***	.05	.02	.42***	.23***	−.28***	−.22***	.16***	.14**	.32***	.17***	.19***	−.28***	.04	−.10*	.23***	.11**	−.08	-				
24. Procedural justice	−.40***	−.45***	.13**	.01	.15***	.02	.04	.32***	.19***	−.26***	−.17***	.10*	.15***	.43***	.21***	.26***	−.23***	.08*	−.06	.23***	.06	−.01	.55***	-			
25. Interactional justice	−.40***	−.46***	−.11**	−.01	−.10*	−.01	.02	.17***	.05	−.24***	−.04	.12**	.23***	.43***	.31***	.31***	−.15***	−.12**	.04	.23***	.05	−.03	.26***	.30***	-		
26. Justice of colleagues	−.19***	−.24***	−.05	.03	−.02	−.05	.06	.09*	.06	−.06	.17***	.19***	.26***	.11**	.22***	.27***	−.02	−.10*	.15***	.13**	.02	−.01	.01	.04	.16***	-	
27. Justice of citizens	−.19***	−.18***	.18***	.02	.16***	−.01	.05	.09*	.09*	−.06	.05	.16***	.12**	.19***	.11**	.20***	−.09*	−.04	.13**	.16***	.14**	−.04	.16***	.14**	.08	.24***	-
28. Organizational identification	−.41***	−.53***	.04	.07	.05	−.01	.01	.11**	.10*	−.14**	.01	.20***	.26***	.29***	.27***	.49***	−.12**	−.04	.03	.25***	.07	−.06	.30***	.37***	.32***	.15***	.16***

^*^p < .05; **p < .01; ***p < .001

## Results

### Descriptive Statistics and Bivariate Correlations

The descriptive statistics for all variables are given in Table 1. The police officers had significantly higher levels of disengagement (*M* = 3.33, *SD* = 0.86) than exhaustion (*M* = 3.25, *SD* = 0.80, *t*(572) =  − 2.70, *p* < 0.01).

First, we examined the pattern of correlations between all the variables under study (Tables 2 and 3). In general, we found that exhaustion and disengagement significantly correlated with most of our predictors: they were both significantly and negatively correlated with all justice perceptions and organizational identification, all the psychosocial protective factors considered, as well as self-care, and higher income. Moreover, exhaustion and disengagement significantly and positively correlated with all the psychosocial risk factors considered, except emotional and cognitive demands^3^ with disengagement. This confirms previous evidence and supports the inclusion of so many variables in our study.

### Multiple Regression Analysis

To avoid multicollinearity issues in the multiple regression analysis, and given that one of the main goals of the paper is to determine the unique impact of justice perceptions and organizational identification on burnout, before selecting the variables for the regression analysis, we first examined the correlation between all the psychosocial factors considered and the justice perceptions and organizational identification measures (Table 2). We found that some of the psychosocial factors were either highly correlated among each other or strongly correlated with one or more measures of justice perceptions or organizational identification. Therefore, we decided not to include the following psychosocial factors in the regression analysis: rewards, social support from supervisor, justice and respect, leadership quality, trust regarding management, social support from colleagues, and social community at work. Furthermore, from the correlations presented in Table 2, we can consider that procedural justice and interactional justice are proxies of the variable support from supervisor, justice and respect, leadership quality, and trust regarding management, whereas justice of colleagues is a proxy of social support from colleagues and social community at work. Table 3 presents the correlations of variables that will be considered potential predictors of burnout.

Furthermore, because we had so many potential variables to be included in the regression, we also reflected about how much this would affect the power of our models. Harris (1985) considers that for regression equations using six or more predictors, an absolute minimum of 10 participants per predictor variable is appropriate, and with approximately 30 participants per variable, which is the case of the present sample, it is possible to detect a small effect size.

After selecting the variables whose analysing the pattern of correlations between the variables under study (Table 3), multiple regression analyses were performed for each burnout dimension (Table 4). The variables were only entered in the regression if they significantly correlated with that burnout dimension: years of professional experience, income, and shift work; quantitative demands, emotional and cognitive demands, influence at work, possibilities for development, predictability, role-clarity, and meaning of work and work pace; affective and cognitive empathy, self-care, religiosity, and political orientation; and distributive, procedural, and interactional justice, justice of colleagues, justice of citizens, and organizational identification.

**Table 4:** Multiple regressions predicting exhaustion and disengagement

	**Exhaustion**	**Disengagement**
	*β*	*β*
Years of professional experience	.12***	–
Income	−.01	−.02
Shift work	−.01	−.03
Quantitative demands	.23***	.07*
Emotional and cognitive demands	.16***	–
Influence at work	−.10**	−.02
Possibilities for development	−.01	−.06
Predictability	−.01	−.10**
Role-clarity	−.06	.03
Meaning of work	−.15***	−.34***
Work pace	.03	.03
Affective empathy	.16***	.09**
Self-care	−.16***	−.03
Religiosity	–	−.03
Political orientation	.04	–
Distributive justice	−.08*	−.10**
Procedural justice	−.11**	−.13**
Interactional justice	−.08*	−.14***
Justice of colleagues	−.04	−.06
Justice of citizens	−.05	−.01
Organizational identification	−.11**	−.18***
**Constant**	3.88	6.21
**R**^**2**^	.55	.58

For all measures, scores were computed by averaging across items, with higher scores indicating stronger endorsement of the construct. For gender, 1 indicates “male” and 2 “female”

*β* Standardized coefficients

^*^p < .05; **p < .01; ***p < .001

In exhaustion, 55% of the variance was predicted by years of professional experience (beta = 0.12), quantitative demands (beta = 0.23), emotional and cognitive demands (beta = 0.16), influence at work (beta = − 0.10), meaning of work (beta = − 0.15), affective empathy (beta = 0.16), self-care (beta = − 0.16), distributive justice (beta = − 0.08), procedural justice (beta = − 0.11), interactional justice (beta = − 0.08), and organizational identification (beta = − 0.10).

More years of professional experience, higher quantitative demands, emotional and cognitive demands, and affective empathy significantly predicted higher exhaustion. Higher influence at work, meaning of work, self-care, distributive, procedural and interactional justice, and organizational identification significantly predicted lower exhaustion.

For disengagement, 58% of the variance was predicted by quantitative demands (beta = 0.07), predictability (beta = − 0.10), meaning of work (beta = − 0.34), affective empathy (beta = 0.09), distributive justice (beta = − 0.10), procedural justice (beta = − 0.13), interactional justice (beta = − 0.14), and organizational identification (beta = − 0.18).

Higher quantitative demands and higher affective empathy significantly predicted higher disengagement. A perception of higher predictability, meaning of work, distributive justice, procedural justice, interactional justice, and organizational identification significantly predicted lower disengagement.

The results showed a consistent impact of quantitative demands; meaning of work; affective empathy; distributive, procedural, and interactional justice; and organizational identification on both burnout dimensions.

## Discussion

This research aimed to conduct a thorough investigation of risk and protective factors for burnout in police officers in a single study. We explored a wide range of psychosocial risk factors, individual variables that have been previously found to be associated with burnout, organizational justice, and organizational identification, and we compared their predictive and unique impact on burnout of police officers.

The results clearly confirm the relevance of our aim. Almost all the predictors correlated with burnout and generally confirmed the findings of previous research with police officers and other professional groups. However, when these predictors were considered together, only a few had a unique impact on both burnout dimensions. These were organizational justice (distributive, procedural, and interactional), organizational identification, and meaningful work as protective factors, with quantitative demands and affective empathy as risk factors. These results assume a particular significance because we considered the impact of many other possible confounds.

Our results highlight the importance of these factors for burnout prevention in police officers and suggest that interventions should focus on these variables rather than on those that affected only one of the burnout dimensions. These were emotional and cognitive demands, influence at work, self-care for preventing exhaustion, and predictability for preventing disengagement.

The results of the present study also have theoretical implications. They suggest that researchers should be cautious when building causal models based on associations obtained in correlational studies, without previously assessing their unique impact on studies considering multiple potential predictors. Some variables may, in fact, appear important predictors just because of their correlation with burnout, but when entered in the regression with other predictors, they may lose their impact. This is what happened, for example, with shift work. Shift work has been considered an important predictor of burnout (Cheng and Cheng 2016; Wisetborisut et al. 2011), and in our study, it correlates significantly with both exhaustion and disengagement. However, when entered in the regression with other predictors, it was no longer a significant predictor.

One interesting issue to notice is the impact of all the dimensions of organizational justice on both exhaustion and disengagement. This highlights that all the three organizational justice dimensions considered (distributive, procedural, interactional) are unique protectors of burnout. Therefore, they should all be considered in programmes to prevent and/or reduce burnout in police officers.

Besides this, in our sample, procedural and interactional justice are not as highly correlated as in samples of other professionals (e.g. physicians and nurses, Correia and Almeida 2020). One possible explanation is related with the characteristics of highly hierarchical and bureaucratic police organizations. In such organizations, the professionals may make a distinction more easily between the procedure that the commander has to follow and the quality of the interpersonal treatment the commander gives them. Additionally, future studies should investigate if the two subcomponents of interactional justice (interpersonal justice and informational justice) have different unique impacts on each of the burnout dimensions.

In sum, based on the results of the present study, we strongly recommend that top managers and those throughout the police command hierarchy should address justice issues in the organization, such as a fair and respectful leadership, that give a voice to their members, treat them with consideration and respect, and reward them fairly (Thomas 2022; Tyler et al. 2015). Furthermore, transparent processes for adjudicating complaints and discipline should be implemented.

It is also very important to reinforce the mechanisms promoting the identification of police officers with their organization and their professional role (Bhowmick and Mulla 2021; Goltz and Smith 2014; Schaible 2018). In addition to the positive outcomes of identification as a police officer already mentioned (e.g. better performance, DeCarufel and Schaan 1990; more supportive attitudes towards progressive policing, e.g. Haarr 1997), our results also showed that organizational identification can protect police officers from burnout.

One possible related area between organizational identification and well-being is a mediation through work engagement and its constituent dimensions (vigour, dedication, and absorption; Karanika-Murray et al. 2015). This could be a future avenue of research into police organizations.

Importantly, the meaning of work’s importance is in line with previous research showing its role as a protective factor for burnout in police officers (Pines and Keinan 2005). These concepts should be included in undergraduate training and also further developed in the workplace.

For empathy, the results showed that cognitive empathy is not associated with burnout, but affective empathy is a risk factor for both exhaustion and disengagement. A similar pattern was found in a study of human health care professional burnout (Correia and Almeida 2020), as well as in animal health care providers (Varela and Correia 2023), and runs counter to the hypothesis of empathy as a burnout protective factor (Zenasni et al. 2012). It also highlights the importance of distinguishing between affective and cognitive empathy in studies of burnout predictors. Consequently, given that empathy is an important social skill for police officers (Bloksgaard and Prieur 2021) and cognitive empathy is not associated with burnout, our results suggest that cognitive empathy can be actively promoted in the selection and training of police officers, with no risk of making police officers more susceptible to burnout.

In sum, promoting meaningful work, organizational justice (distributive, procedural, and interactional justice), and organizational identification in police organizations may be key for protecting police officers from the risk of burnout or preventing it from occurring in the first place. However, we should not devalue the responsibility of organizations to reduce risk factors to which police officers are subject, and in the case of police officers, this proved to be quantitative demands. Therefore, although protective factors may be reinforced, excessive workload is an important risk factor and decreasing it continues to be central to burnout prevention.

It is vital that police officer burnout is addressed because of its important consequences for the well-being of these professionals, as well as for the efficacy of the organizations in which they work and for maintaining the social systems’ security. Illegitimate police action, possibly caused by burnout syndrome, may turn the public against the police institution as a whole (e.g. Tyler and Folger 1980).

### Limitations

Our study has however some limitations. A first limitation is related with the correlational design of this study, which restricts the nature of the conclusions that can be drawn about the causal relations among variables. A second limitation refers to the convenience nature of our sample that, despite being large, is probably not representative of the GNR police officer population, nor of the population of police officers in Portugal or even less across the world. There are two more limitations related with the fact that all predictors and outcome variables were self-reported: self-report biases (Donaldson and Grant-Vallone 2002) and common-method biases (Podsakoff et al. 2003). However, considering that our variables are very much related with the subjective perception of the individuals, self-reports produce the best measure, even considering these two limitations associated with their use.

## Conclusion

Our study clearly identifies key variables that are unique predictors of burnout. They are quantitative demands and affective empathy as burnout risk factors and meaningful work, organizational justice (distributive, procedural, and interactional justice), and organizational identification as burnout protective factors. We strongly recommend that these findings are disseminated amongst researchers, policymakers, and police professionals, as they could be the basis for decision-making and specific prevention programmes in order to reduce demands and strengthen police officer resources. Future research should overcome the fragmented research approach that has been prevalent so far and that consists of studying the impact of separate variables found to be correlated with burnout. A more integrated perspective should be adopted that simultaneously considers the main predictors that can be the basis for burnout prevention.

## Data Availability

The datasets presented in this article are not publicly available because the participants of this study did not give the permission for their data to be shared publicly. However, the datasets can be available under request directed to Isabel.Correia@iscte-iul.pt.

## Footnotes

1 Some of the items have been adapted to the specific context of the military police officers with the collaboration of the Trade Union leaders. For instance, instead of “My superior”, the term used was “My commander”.

2 Again, some of the items were adapted to better fit the specific context of the police officers of the Guarda Nacional Republicana.

3 We decided to join emotional demands (1 item) and cognitive demands (3 items) in one variable of “emotional and cognitive demands” because a factorial analysis of these four items with varimax rotation extracted only one factor explaining 51.91% of the total variance. The variable “emotional and cognitive demands” is the mean of the four items.
